# A new species of *Carcinonemertes*, *Carcinonemertes conanobrieni* sp. nov. (Nemertea: Carcinonemertidae), an egg predator of the Caribbean spiny lobster, *Panulirus argus*

**DOI:** 10.1371/journal.pone.0177021

**Published:** 2017-05-05

**Authors:** Lunden Alice Simpson, Louis John Ambrosio, J. Antonio Baeza

**Affiliations:** 1 Department of Biological Sciences, Clemson University, Clemson, South Carolina, United States of America; 2 Smithsonian Marine Station at Fort Pierce, Fort Pierce, Florida, United States of America; 3 Departamento de Biología Marina, Facultad de Ciencias del Mar, Universidad Católica del Norte, Larrondo, Coquimbo, Chile; Evergreen State College, UNITED STATES

## Abstract

A new species of nemertean worm belonging to the genus *Carcinonemertes* is described from egg masses of the Caribbean spiny lobster *Panulirus argus* from the Florida Keys, Florida, USA. This is the first species of *Carcinonemertes* reported to infect *P*. *argus* or any other lobster species in the greater Caribbean and western Atlantic Ocean. *Carcinonemertes conanobrieni* sp. nov. varies in body color from a translucent white to a pale orange, with males ranging in total body length from 2.35 to 12.71 mm and females ranging from 0.292 to 16.73 mm. Among the traits that separate this new species from previously described species in the genus *Carcinonemertes* are a relatively wide stylet basis, minimal sexual size dimorphism, and a unique mucus sheath decorated with external hooks. Also, juvenile worms were found to encyst themselves next to lobster embryos and female worms lay both long strings of eggs wound throughout the lobster’s setae as well as spherical cases that are attached to lobster embryos. The stylet length and stylet basis remain unchanged throughout ontogeny for both male and female worms. Maximum likelihood and Bayesian inference phylogenetic analyses separated this newly described species from all other species of *Carcinonemertes* with available COI sequences. *Carcinonemertes* spp. are voracious egg predators and have been tied to the collapse of various crustacean fisheries. The formal description of this new species represents the first step to understand putative impacts of this worm on the population health of one of the most lucrative yet already depressed crustacean fisheries.

## Introduction

Marine ecosystems are one of the most heavily used and valuable natural systems worldwide [1] providing globally relevant ecosystem services (e.g., shoreline protection, water filtration, nursery grounds, feeding grounds to commercially important fishes–[2, 3]). At the same time, these complex and well-interconnected marine systems are vulnerable to both natural and human perturbations [4]. Global climate change and increasing ocean temperatures, among others, have been shown to impact the survival, growth, and health of marine organisms [5] and periods of thermal stress have led to disease outbreaks [6, 7]. As ocean temperature rises many marine organisms, including pathogens, are shifting towards the poles [8, 9] leading to changes in the interactions between hosts and pathogens. This in turn, has the potential to lead to changes in the frequency and severity of disease events ([7]; reviewed in [3]). One such affected area, the wider Caribbean region, is considered a disease hot spot characterized by the rapid emergence of a variety of new and virulent diseases, and typically at a higher prevalence than in other regions (reviewed in [10]). Over the past 25 years the significant warming of the Caribbean basin [11] has coincided with coral bleaching events, disease emergence, and an increasing frequency of infectious disease outbreaks [12–14; 3].

Spiny lobsters, including the Caribbean spiny lobster, *Panulirus argus*, have been shown to play host to a variety of marine diseases and pathogens, including some newly emergent diseases (reviewed in [15, 16]). *Panulirus argus* Virus 1 (PaV1) is one such emergent disease infecting *P*. *argus* and was first reported in 2004 by Shields and Behringer [17]. *Panulirus argus* is also know to host multiple species of bacteria [18–20], helminths [21], and crustaceans [22]. Though *P*. *argus* has not yet been reported to host a *Carcinonemertes* sp. worm, other spiny lobsters have. Examples of spiny lobster species that are infected by *Carcinonemertes* spp. include: *Panulirus interruptus* (infected by *Carcinonemertes wickhami*) [23], *Panulirus cygnus* (infected by *Carcinonemertes australiensis*) [24], and *Jasus edwardsii* [16].

*Carcinonemertes* worms belong to the nemertean worm family Carcinonemertidae which also includes the genus *Ovicides*. Members of *Carcinonemertes* may be separated from *Ovicides* in that they possess only a single stylet with no accessory pouches and are gonochoric, while *Ovicides* is distinguished by accessory stylets and species can be either gonochoric or hermaphroditic [25, 26]. Members of this family are considered symbiotic egg predators of decapod crustaceans. To date, there are 16 described species of *Carcinonemertes*, and 5 described species of *Ovicides* found in association with approximately 70–75 recorded host species [26–29] with most occurring on cancrid, portunid, and xanthoid crabs; though two species have been reported on panulirid lobsters [24, 25]. Members of this family vary in terms of host specificity, with some species inhabiting a single host species (*C*. *errans* [30]; and *O*. *juliaea* [25]) while others are reported on more than a dozen decapod species of crab (*C*. *c*. *carcinophila* and *C*. *c*. *imminuta*) [26, 27]. These worms are often overlooked because they usually show low prevalence in host populations, they live in cryptic locations on the host bodies, and/or they typically only mature on ovigerous hosts, meaning in some instances they may be observed only seasonally [26, 31].

During an investigation into the active parental care and reproductive performance of the Caribbean spiny lobster, *Panulirus argus*, in the summer of 2015, we noticed the presence of a nemertean worm in the egg mass of a few female lobsters [32]. Upon further inspection, we concluded that this nemertean belonged to the genus *Carcinonemertes*. Here we describe *Carcinonemertes conanobrieni*, a new species in the family Carcinonemertidae found in the broods of *P*. *argus*. Distinctive morphological characters and some aspects of the life history of this new species are discussed and presented against those of other members within the genus.

## Material and methods

### Collection of host and parasite specimens

Caribbean spiny lobsters, *Panulirus argus*, were collected from July 10^th^ to July 19^th^, 2016 from two coral reefs (5–10 m depth) along the Florida reef tract. Collection was possible through a Special Activity License through the Florida Fish and Wildlife Conservation Commission (SAL-15-1674A-SR). The first collection site was approximately 5 km off of Long Key, Florida at Tennessee Lighthouse Reef (24.7707 N, -80.7615 W) and the second site was approximately 5 km off of Duck Key, Florida at Critter Ridge Reef (24.7325 N, -80.9121 W). At each locality, gravid female lobsters were gently captured by hand (with the aid of a tickle stick and hand net) while SCUBA diving, and then transported alive in the R/V *Soledad* to a temporal laboratory in Long Key, Florida. Lobsters were maintained alive in two 416.5 liter cattle tanks with bubbling aerators until dissection.

Next, pleopods were removed from gravid females, and all embryos were gently stripped away from the pleopods and placed into Petri dishes filled with seawater using microforceps. The embryo masses were then inspected for the presence of nemerteans under either a Leica S8AP0 stereoscope or a Wild M5-97874 dissecting scope. The remainder of the host lobster anatomy (including abdomen, pleopods, eye orbitals the joints of walking legs, gills and branchial chamber) was also visually inspected to determine the presence of nemerteans using the same stereoscopes.

Nemerteans collected from lobsters were placed in Petri dishes filled with seawater until the moment of taking measurements, photographs, and notes on morphological characters. Nemerteans were first relaxed in a 1:1 solution of 1M MgCl_2_ (prepared with distilled water) and seawater for 1–5 min., after which, length and width of the body, the distance between the eyes, and the distance from the eyes to the tip of the head were determined with the help of a micrometer slide, Leica S8AP0 Stereoscope, and Leica camera MC170 HD. Measurements of internal features were made with the help of an ocular micrometer in a compound microscope after covering the worms with a coverslip. The holotype and paratype specimens were preserved in a 7% formalin-seawater solution. Other specimens were fixed in 99% EtOH solution for genetic analysis.

### Phylogenetic position of the new species

Total genomic DNA was extracted from whole specimens of the nemertean worm using the QIAGEN^®^ DNeasy^®^ Blood and Tissue Kit following the protocol recommended by the manufacturer. The polymerase chain reaction (PCR) was used to amplify the target region of the mitochondrial cytochrome *c* oxidase subunit I (COI) gene. For the amplification of COI, we used the primers LCO1490 (5'-ggt caa caa atc ata aag ata ttg g-3') and HCO2198 (5'-taa act tca ggg tga cca aaa aat ca-3') [33]. Standard PCR 25-μl reactions (12.5-μl GoTaq^®^ MasterMix (Promega), 2.5-μl each of the two primers, and 7.5-μl of DNA template) were performed on a C1000 Touch^™^ Thermal Cycler (BIORAD, Hercules, CA, USA) under the following conditions: initial denaturation at 95°C for five minutes followed by 35 cycles of 95°C for 1 min, 51°C for 1 min, and 72°C for 1 min, followed by chain extension at 72°C for 10 minutes. The post-PCR products were purified with ExoSapIT (a mixture of exonuclease and shrimp alkali phosphate, Amersham Pharmacia) and then sent for Sanger Sequencing to Clemson University’s Genomics Institute (CUGI—Clemson University, Clemson, South Carolina), which is equipped with an ABI Prism 3730xl Genetic Analyzer (Applied Biosystems automated sequencer). All sequences were confirmed by sequencing both strands and a consensus sequence was obtained from the two strands using the software Sequencer (Gene Codes Corp.).

A total of 9 other species of *Carcinonemertes* were used as ingroup terminals for molecular comparisons with our new species, with 4 other species of ribbon worm, *Ovicides* sp., *Nipponnemertes punctatula*, *Nipponnemertes bimaculata*, and *Nipponnemertes pulchra* included in the analysis as outgroup terminals. The species of *Carcinonemertes* above were chosen as they represented the totality of COI sequence data available. *Ovicides paralithodis* was chosen as an outgroup terminal because it is the only other genera that belongs to the family Carcinonemertidae. *Nipponnemertes bimactulata*, *Nipponnemertes punctatula* and *Nipponnemertes pulchra* were also chosen as outgroup species based on recent phylogenetic studies that placed *Nipponnemertes* as sister to *Carcinonemertes* in the clade monostilifera [34]. All COI sequences, outside the ones generated by us, were retrieved from Genbank.

Sequence alignment was conducted using Multiple Sequence Comparison by Log-Expectation in MUSCLE [35] as implemented in MEGA 6 [36]. The alignment of the COI gene fragment had no indels and was unambiguous.

The dataset was first analyzed with the software jModelTest2 [37] which compares different models of DNA substitution in a hierarchical hypothesis-testing framework to select a base substitution model that best fits the given data [38]. The optimal model found by jModelTest2 (GTR) was implemented in MrBayes [39] for Bayesian Inference (BI) analysis and in PhyML for maximum likelihood (ML) analysis (PhyML may be accessed with: http://www.atgc-montpellier.fr/phyml/) [40]. Missing data were designated as a “?” in the alignment.

All the parameters used for the ML analysis were those of the default options in PhyML. For BI, unique random starting trees were used in the Metropolis-coupled Markov Monte Carlo Chain (MCMC) (see [39, 41]). This analysis was performed for 6,000,000 tree generations. Visual analysis of the log-likelihood scores against the generation time indicated that the log-likelihood values reached a stable equilibrium before the 100,000^th^ generation. Thus, a burn-in of 1,000 samples was conducted and every 100^th^ tree was sampled from the MCMC analysis obtaining a total of 60,000 trees and a consensus tree with the 50% majority rule was calculated for the last 59,900 sampled trees. The robustness of the ML tree topology was assessed by 2,000 bootstrap iterations of the data. Support nodes for the BI tree topology were obtained by posterior probability.

### Correlation analyses

We performed classical correlation analyses between stylet characteristics (stylet length, basis length, and stylet:basis ratio) and maximum body length for both male and female *C*. *conanobrieni* using JMP Pro 12 Software [42]. We also used JMP Pro 12 software [42] in ANCOVA analyses that compared the stylet structures between the sexes. In these ANCOVAs, sex was the independent variable, the stylet structure measurement was the dependent variable, and worm maximum body size was set as the covariate.

### Nomenclatural acts

The electronic edition of this article conforms to the requirements of the amended International Code of Zoological Nomenclature, and hence the new names contained herein are available under that Code from the electronic edition of this article. This published work and the nomenclatural acts it contains have been registered in ZooBank, the online registration system for the ICZN. The ZooBank LSIDs (Life Science Identifiers) can be resolved and the associated information viewed through any standard web browser by appending the LSID to the prefix “http://zoobank.org/”. The LSID for this publication is: urn:lsid:zoobank.org:pub:9D73818B-E952-4494-BF6F-4AF4FF38C7E4. The electronic edition of this work was published in a journal with an ISSN, and has been archived and is available from the following digital repositories: PubMed Central, LOCKSS.

## Results

### Diagnosis—Family Carcinonemertidae Sumner et al., 1913

The following diagnosis of the family Carcinonemertidae is taken from Humes [27] and modified by Shields et al. [43]: Members are monostiliferous hoplonemerteans living as symbiotic egg predators on the gills, beneath the abdomen, on the apodemes and axillae, and in the egg masses of decapod crustaceans. They possess a reduced proboscis and a short, poorly developed rhynchocoel. The lateral nerves lie internal to well-developed submuscular glands. Cephalic glands well developed, with cephalic muscle fibers present. Missing cerebral sensory organs, and possess 2 ocelli. Takakura’s duct system is present in males. Internal fertilization and ovovivparity commonly occur; extensive development of spermatozoa and ova. Most species secrete and reside within, temporarily, a mucus sheath that is attached to the setae on the pleopods and hairs of endopodites of ovigerous decapods. Embryos hatch as hoplonemertean planuliform larvae.

### Diagnosis—Genus *Carcinonemertes* Coe, 1902

The following diagnosis of the genus *Carcinonemertes* is taken from Coe [44] and modified by Santos et al. [45] and Sadeghian and Santos [27]: Members are nemerteans living as symbiotic egg predators on numerous species of Crustacea. With a reduced proboscis and a short, poorly developed rhynchocoel; armed with a central stylet and basis only; no lateral pouches or reserve stylets. No distinct muscular layers in the body wall, no distinct nerves, and without a thickened glandular epithelium. Missing cerebral sensory organs, and possess 2 ocelli. Cephalic glands massively developed. Internal and external fertilization and both occur.

### Diagnosis–*Carcinonemertes conanobrieni* sp. nov. urn:lsid:zoobank.org:pub:9D73818B-E952-4494-BF6F-4AF4FF38C7E4

Body color varies from white to pale orange. The anterior end of the body can be either rounded or pointed. The posterior end can be either rounded or pointed. Worms are filiform in shape and range from 0.292 mm to 16.73 mm in length. Males are not significantly smaller than females. No accessory stylets present. Ovaries arranged in a single row on either side of the intestine. Adult worms can be found free roaming through the host’s egg mass and may produce mucus sheaths that wind through the pleopod setae of gravid female hosts.

### Material examined

Seventeen females, 15 males, and 4 larvae. Holotype: female taken from the egg mass of an adult Caribbean spiny lobster *Panulirus argus*. Type locality: holotype female was taken from a gravid female lobster caught along Tennessee Lighthouse Reef off of Long Key, Florida in July 2016. Paratype specimens were taken from gravid female lobsters captured on either Critter Ridge Reef off Duck Key, Florida or Tennessee Lighthouse Reef off of Long Key, Florida in July 2016. Holotype female (USNM 1422303) and paratypes of both sexes (USNM 1422304—USNM 1422330) have been deposited in the Department of Invertebrate Zoology, National Museum of Natural History, Smithsonian Institution, Washington, DC.

### Etymology

This new species of *Carcinonemertes* is named after the social commentator and comedian Conan O’Brien. The physical similarities between the new species and Mr. O'Brien are remarkable; both exhibit a long and pale soma with slight tints of orange.

### Description

The description of this species is based on living adults and four larvae. Measurements are given in mm as mean ± SD (range, number of specimens observed).

### Female

**Fig 1:**
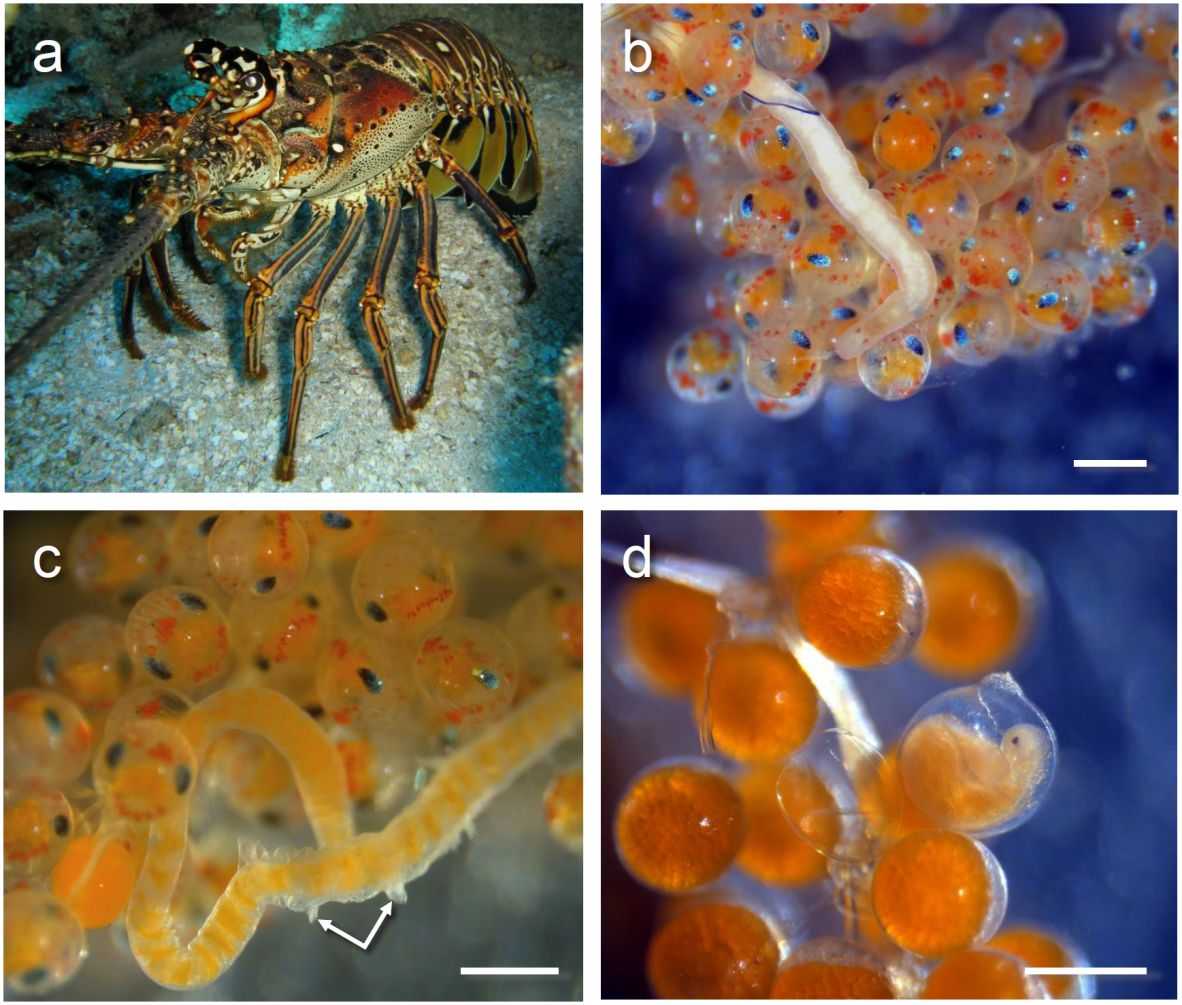
*Panulirus argus* and representative photographs of *Carcinonemertes conanobrieni*. (a) Shows a female *P*. *argus* on a reef in the Florida Keys, the remaining photographs are representative of some of the different ways *Carcinonemertes conanobrieni* may be found within the lobster brood mass [the scale bars in photos b, c, and d all indicate 0.5 mm]. (b) Male *C*. *conanobrieni* free-roaming among late stage lobster embryos. (c) Female *C*. *conanobrieni* partially covered by a mucus sheath with decorative hooks (indicated by arrows) protruding. (d) *C*. *conanobrieni* of undetermined sex encapsulated next to early stage lobster embryos.

**Fig 2:**
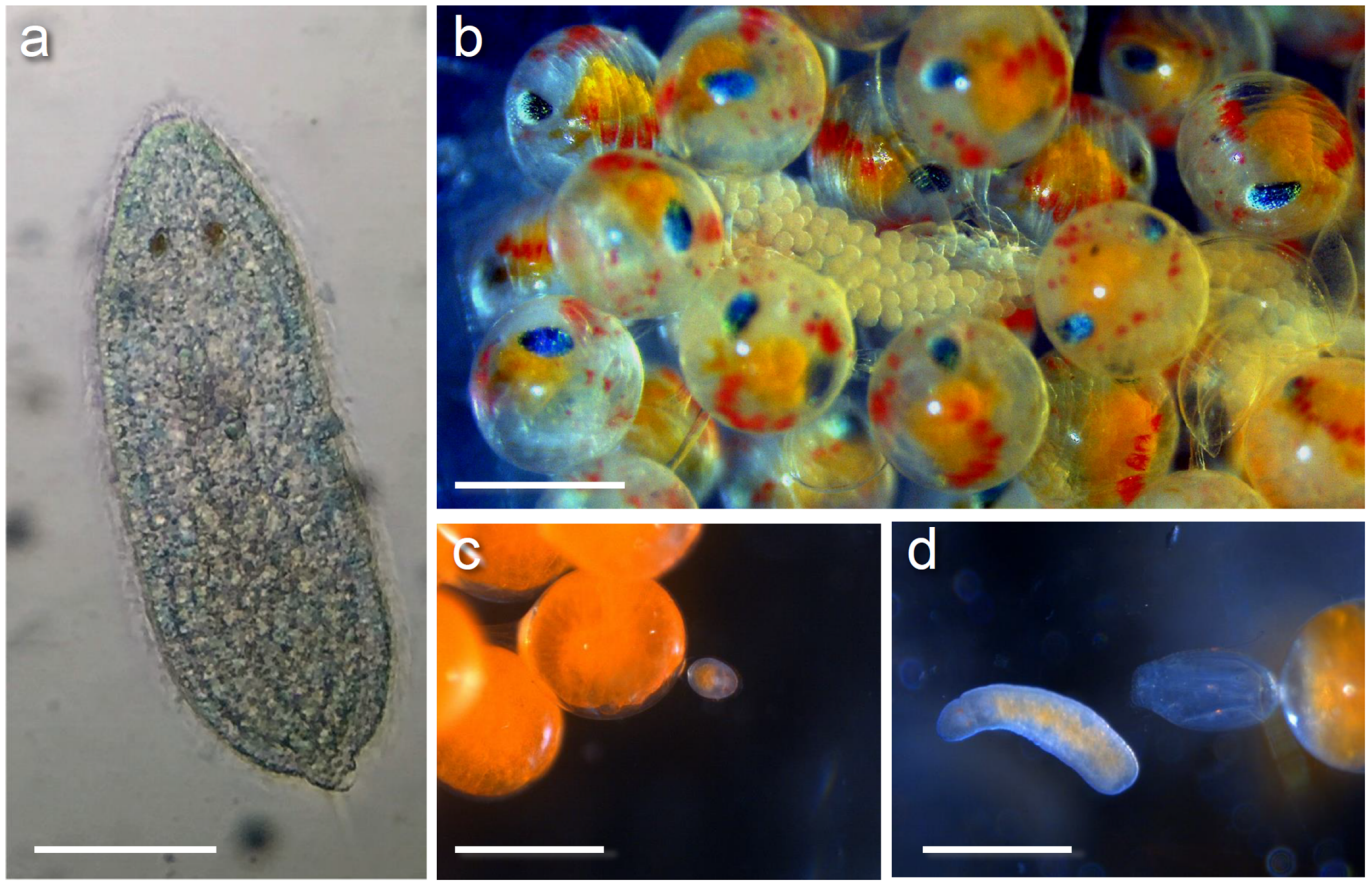
*Carcinonemertes conanobrieni* hoplonemertean larvae, egg cases, and early juveniles. (a) Shows a dorsal view of a hoplonemertean larvae that had been stained with methylene blue for better contrast [scale bar represents 0.05 mm]. (b) A string of *C*. *conanobrieni* embryos wound through late stage lobster embryos [scale bars in b, c, d represent 0.5 mm]. (c) A juvenile *C*. *conanobrieni* encysted next to an early stage lobster embryo. (d) A newly emerged juvenile *C*. *conanobrieni* worm from its cyst attached to a lobster embryo.

**Fig 3:**
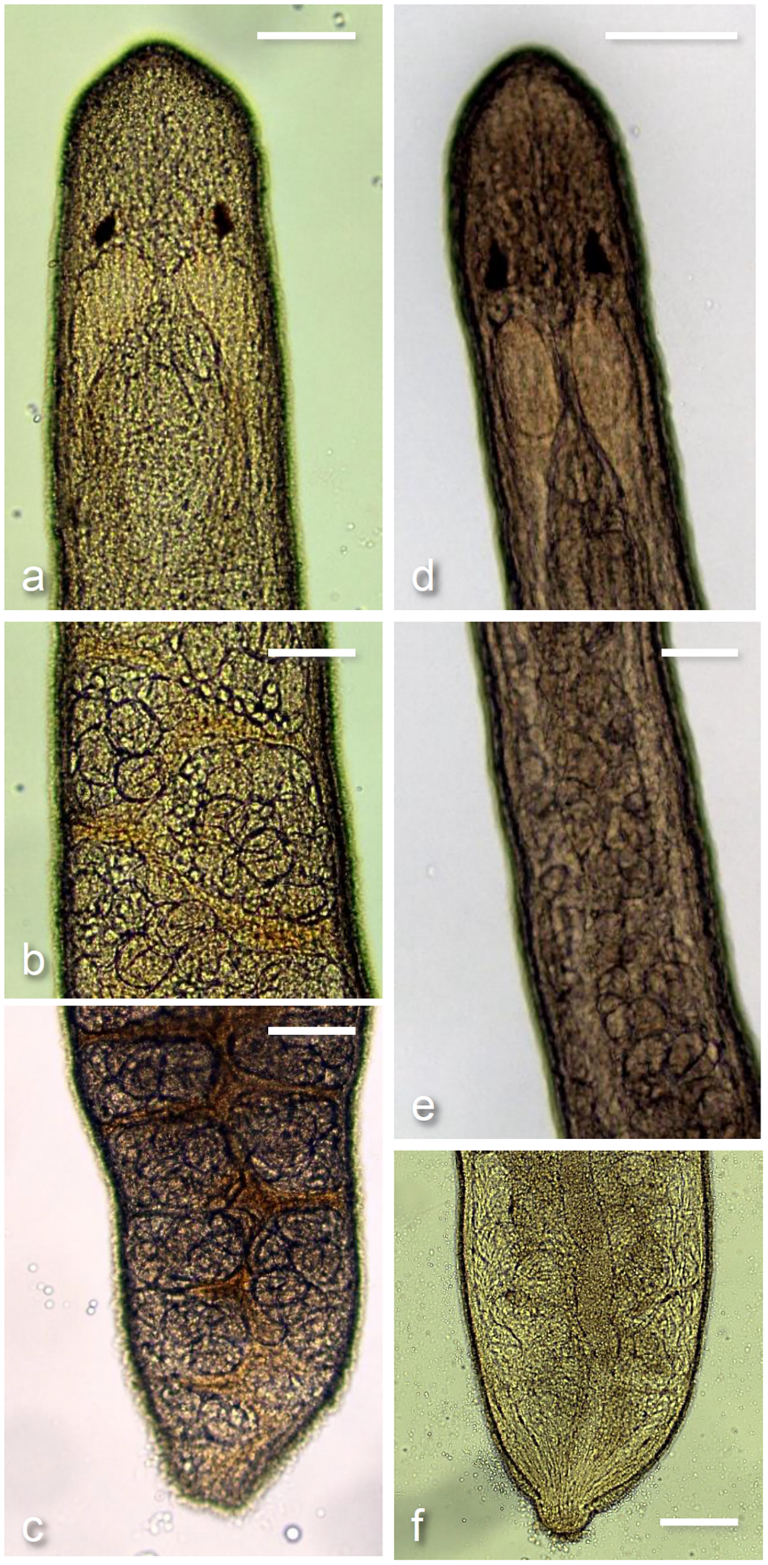
Representative body segments of male and female *Carcinonemertes conanobrieni*. Each vertical set of photos shows sections of the anterior, trunk, and posterior for a female (left) and male (right) *C*. *conanobrieni* [the scale bar in each photograph represents 0.1 mm]. (a) And (d) show the anterior portions of a female and male worm with ocelli, cerebral lobes, and stylet all visible. (b) Shows a section of the trunk of a female *C*. *conanobrieni* with full ovaries separated by the intestinal diverticula, and (e) depict a section of a male’s trunk with testes distributed throughout. (c) Is the posterior end of a female, which has ovaries present for the entire length and (f) is the posterior end of a male with testes stopping just prior to the seminal vesicle (not clearly visible).

**Fig 4:**
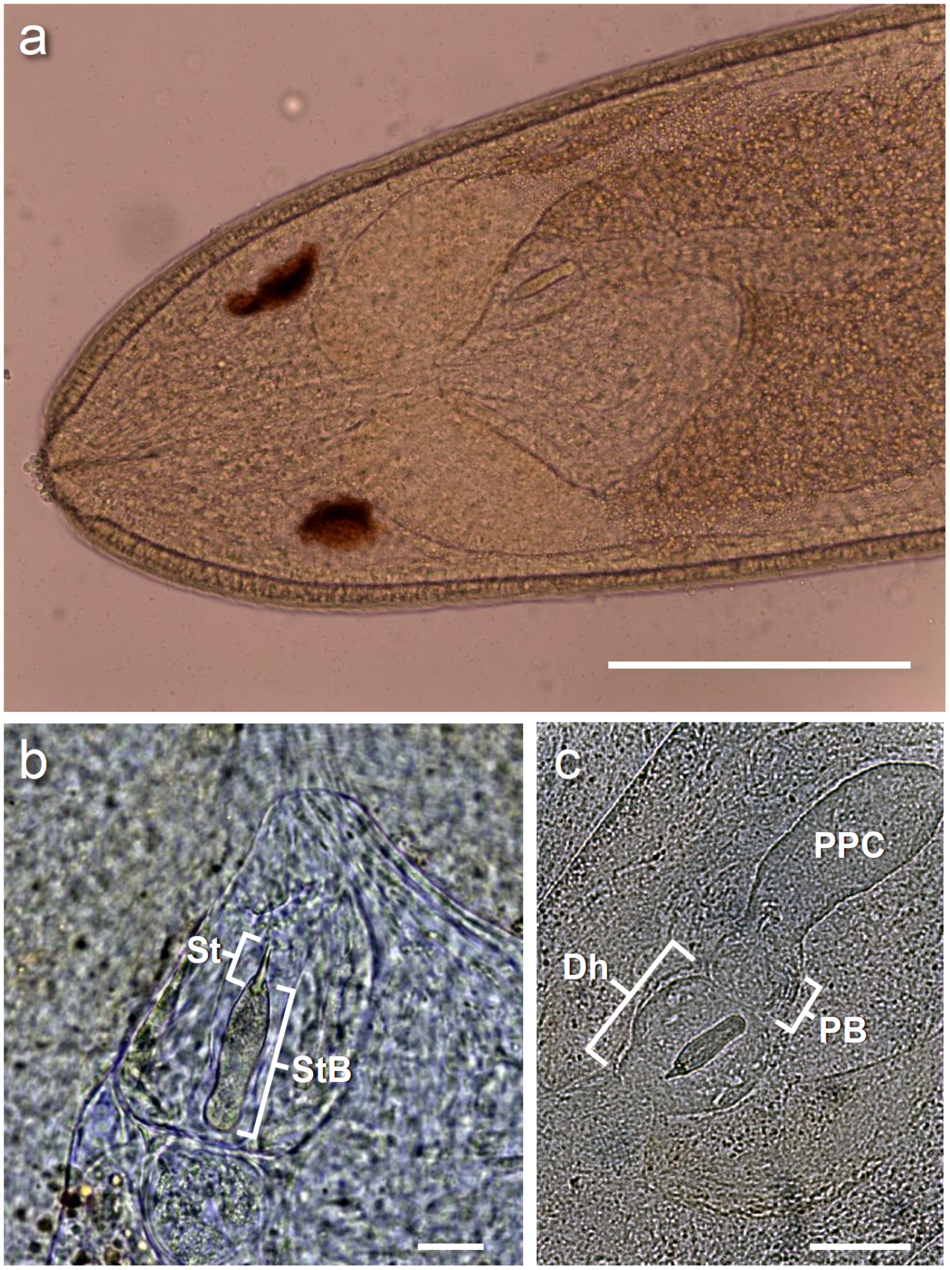
Anterior end of *Carcinonemertes conanobrieni* with a focus on the stylet and surrounding regions. (a) Ventral view of the anterior section of a male *C*. *conanobrieni*; the stylet is positioned just below the right cerebral lobe and is slightly angled [the scale bar represent 0.2 mm]. (b) A slightly angled stylet [St] and stylet basis [StB] [scale bar represent 0.02 mm]. (c) A clear depiction of the stylet, stylet basis, posterior proboscis chamber [PPC], proboscis bulb [PB], diaphragm [Dh], and part of the anterior proboscis chamber.

Body color of specimens varied from a cream to a pale orange. The gut can be either white (empty) to bright orange (full). Gonads are translucent white. Two eyes that range in color from bright orange to a ‘rusty’ red. Eyes are irregular in shape and may be circular, elliptical, or rhomboid; round eyes are the most common shape. Females may be found roaming free among the egg mass of the host, encysted next to host eggs, or in mucus sheaths wound through the host’s pleopods (Figs 1 and 2). Both the anterior end and the posterior end may be rounded or pointed (Fig 3). Dimensions of relaxed worms 6.12 ± 4.32 mm (0.292–16.73 mm, 17) long and 0.540 ± 0.647 mm (0.246–3.02 mm, 17) wide. Single stylet on basis 0.012 ± 0.003 mm (0.008–0.019 mm, 14) long and 0.003 ± 0.001 mm (0.001–0.006 mm, 14) wide. Stylet basis 0.041 ± 0.005 mm (0.033–0.053 mm, 14) long and 0.009 ± 0.002 mm (0.006–0.012 mm, 14) wide. Stylet:basis ratio 0.296 ± 0.078 (0.158–0.429, 14) (Fig 4). No accessory stylets present. Ovaries are arranged in a single row on either side of the intestinal diverticula (Fig 3). All measurements for additional characters used in the species description can be found in Table 1.

**Table 1:** Additional measurements of *Carcinonemertes conanobrieni* sp. nov. used to differentiate this species from other congeneric species. All measurements are given in mm (exceptions include: stylet:basis ratio, a ratio with no units, and the number of ovaries is a direct count). The number in parentheses (#) following the range of measurements indicates the number of specimens analyzed.

Character	Males			Females		
	Mean	Standard Deviation	Range	Mean	Standard Deviation	Range
Body Length	7.03	± 3.41	2.35–12.71 (15)	6.12	± 4.32	0.292–16.73 (17)
Body Width	0.253	± 0.0420	0.157–0.331 (15)	0.540	± 0.647	0.246–3.02 (17)
Eye Length	0.037	± 0.008	0.023–0.050 (15)	0.033	± 0.013	0.019–0.066 (17)
Eye Width	0.027	± 0.007	0.019–0.041 (15)	0.025	± 0.006	0.016–0.040 (17)
Distance: Between Eyes	0.077	± 0.022	0.043–0.111 (15)	0.087	± 0.025	0.054–0.143 (17)
Distance: Eyes to Tip of Head	0.175	± 0.031	0.106–0.229 (15)	0.166	± 0.041	0.083–0.211 (17)
Brain Lobe Length	0.121	± 0.019	0.08–0.155 (15)	0.128	± 0.036	0.074–0.22 (16)
Brain Lobe Width	0.084	± 0.019	0.05–0.11 (15)	0.091	± 0.034	0.053–0.195 (16)
Distance: Top of Brain to Tip of Head	0.211	± 0.043	0.13–0.29 (15)	0.218	± 0.073	0.11–0.345 (16)
Anterior Proboscis Chamber Length	The length continued to the top of the head and could not be accurately determined					
Anterior Proboscis Chamber Width	0.028	± 0.010	0.015–0.048 (10)	0.0246	± 0.008	0.011–0.04 (10)
Diaphragm Length	0.058	± 0.013	0.028–0.073 (15)	0.063	± 0.010	0.051–0.09 (14)
Diaphragm Width	0.050	± 0.012	0.03–0.076 (15)	0.053	± 0.0133	0.029–0.083 (14)
Proboscis Bulb Length	0.027	± 0.007	0.015–0.038 (15)	0.031	± 0.011	0.016–0.053 (14)
Proboscis Bulb Width	0.027	± 0.008	0.03–0.051 (14)	0.041	± 0.013	0.025–0.065 (14)
Posterior Proboscis Chamber Length	0.108	± 0.040	0.07–0.15 (3)	Posterior proboscis chamber measurements were only taken for three male specimens		
Posterior Proboscis Chamber Width	0.039	± 0.010	0.028–0.048 (3)			
Single Stylet Length	0.010	± 0.003	0.006–0.016 (15)	0.012	± 0.003	0.008–0.019 (14)
Single Stylet Width	0.003	± 0.001	0.001–0.006 (15)	0.003	± 0.001	0.001–0.006 (14)
Stylet Basis Length	0.043	± 0.003	0.039–0.048 (15)	0.041	± 0.005	0.033–0.053 (14)
Stylet Basis Width	0.009	± 0.002	0.006–0.013 (15)	0.009	± 0.002	0.006–0.012 (14)
Stylet:Basis Ratio	0.241	± 0.076	0.139–0.407 (15)	0.296	± 0.078	0.158–0.429 (14)
Distance: Tip of Stylet to Tip of Head	0.245	± 0.076	0.125–0.265 (14)	0.251	± 0.088	0.12–0.415 (14)
Number of Ovaries	----			87.4	± 43.6	48–186 (12)
Seminal Vesicle Length	0.408	± 0.188	0.25–0.90 (12)	----		
Distance: First Gonad to Tip of Head	0.554	± 0.169	0.3–0.85 (14)	0.691	± 0.201	0.375–1.09 (15)

### Male

Body color of specimens varied from a translucent white to a cream. The gut can be either white (empty) to bright orange (full). Gonads are translucent white. Two eyes that range in color from bright orange to a ‘rusty’ red. Eyes are irregular in shape and may be circular, elliptical, or rhomboid; round eyes are the most common shape. Males may be found roaming free among the egg mass of the host, encysted next to host eggs, or in mucus sheaths wound through the host’s pleopods (Figs 1 and 2). Both the anterior end and the posterior end may be rounded or pointed (Fig 3). Dimensions of relaxed worms 7.03 ± 3.41 mm (2.35–12.71 mm, 15) long and 0.253 ± 0.0420 mm (0.157–0.331 mm, 15) wide. Single stylet on basis 0.010 ± 0.003 mm (0.006–0.016 mm, 15) long and 0.003 ± 0.001 mm (0.001–0.006 mm, 15) wide. Stylet basis 0.043 ± 0.003 mm (0.039–0.048 mm, 15) long and 0.009 ± 0.002 mm (0.006–0.013 mm, 15) wide (Fig 4). Stylet:basis ratio 0.241 ± 0.076 (0.139–0.407, 15). No accessory stylets present. Seminal vesicle 0.408 ± 0.188 mm (0.25–0.9 mm, 12) long. All measurements for additional characters used in the species description can be found in Table 1.

### Larva

The bodies of larvae are ciliated with both anterior and posterior ciliary tufts (Fig 4). The body shape can be either ovoid (extended) or spherical (contracted). Larvae posses two eyes, orange in color, which may be either circular or elliptical. Dimensions of the larval body are 0.115 ± 0.005 mm (0.113–0.123 mm, n = 4) long and 0.051 ± 0.018 mm (0.043–0.078 mm, n = 4) wide.

### Quantitative soma and body part measurements

**Fig 5:**
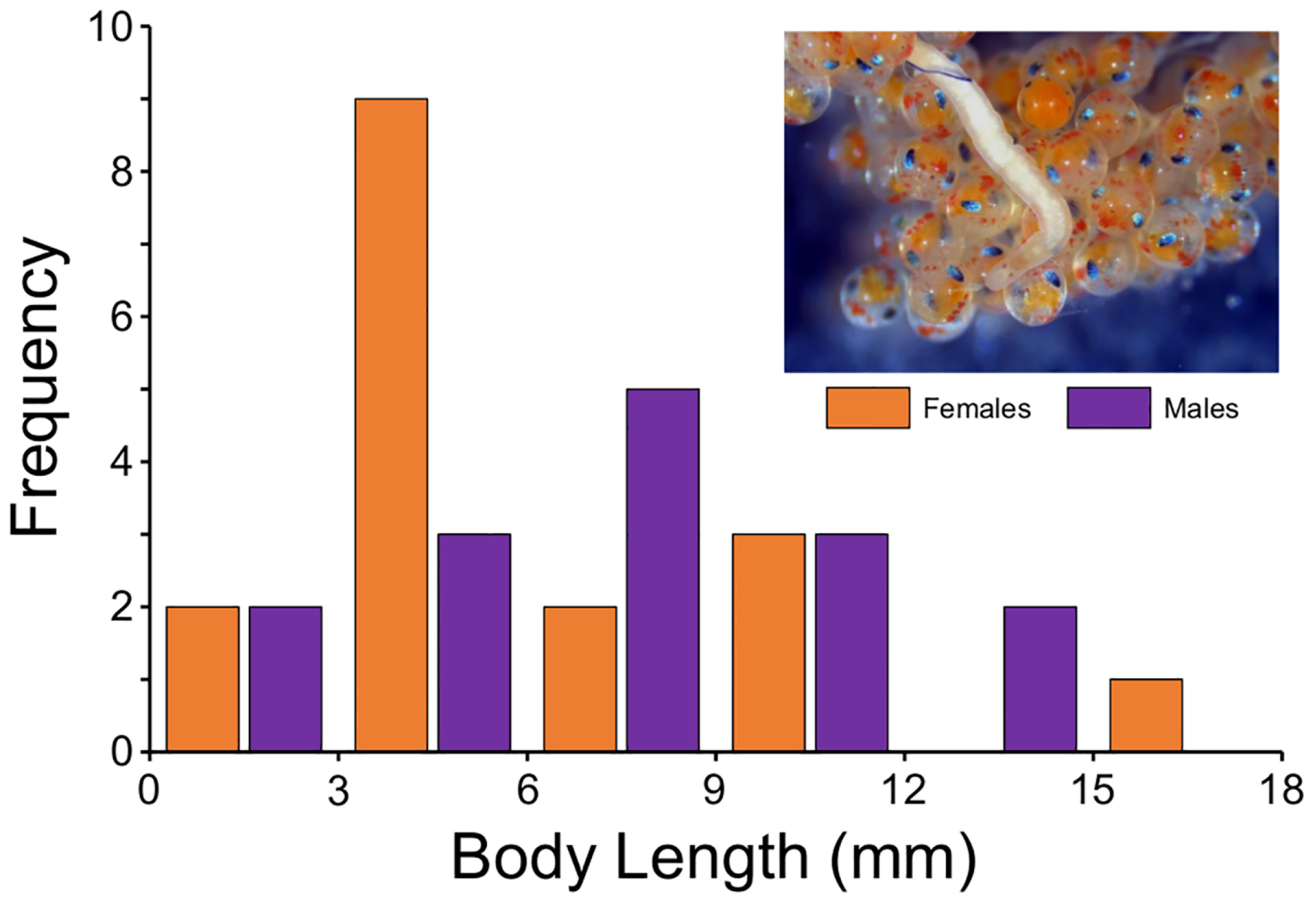
Size-frequency distribution of male and female *Carcinonemertes conanobrieni*. Male body size ranged from 2.35 to 12.71 mm (mean, 7.03 ± 3.41 mm) and female body size ranged from 0.292 to 16.73 mm (mean, 6.12 ± 4.32 mm). In the upper-right is a female *C*. *conanobrieni* within the brood mass of its lobster host.

Male *C*. *conanaobrieni* had a mean body size of 7.03 ± 3.41 mm and ranged in length between 2.35–12.71 mm; female *C*. *conanobrieni* had a mean body size of 6.12 ± 4.32 mm and ranged in length from 0.292–16.73 mm (Fig 5). A t-test showed that there was no significant difference between the mean body size of male and female worms (t = 0.6550, d.f. = 30, P = 0.2587).

**Fig 6:**
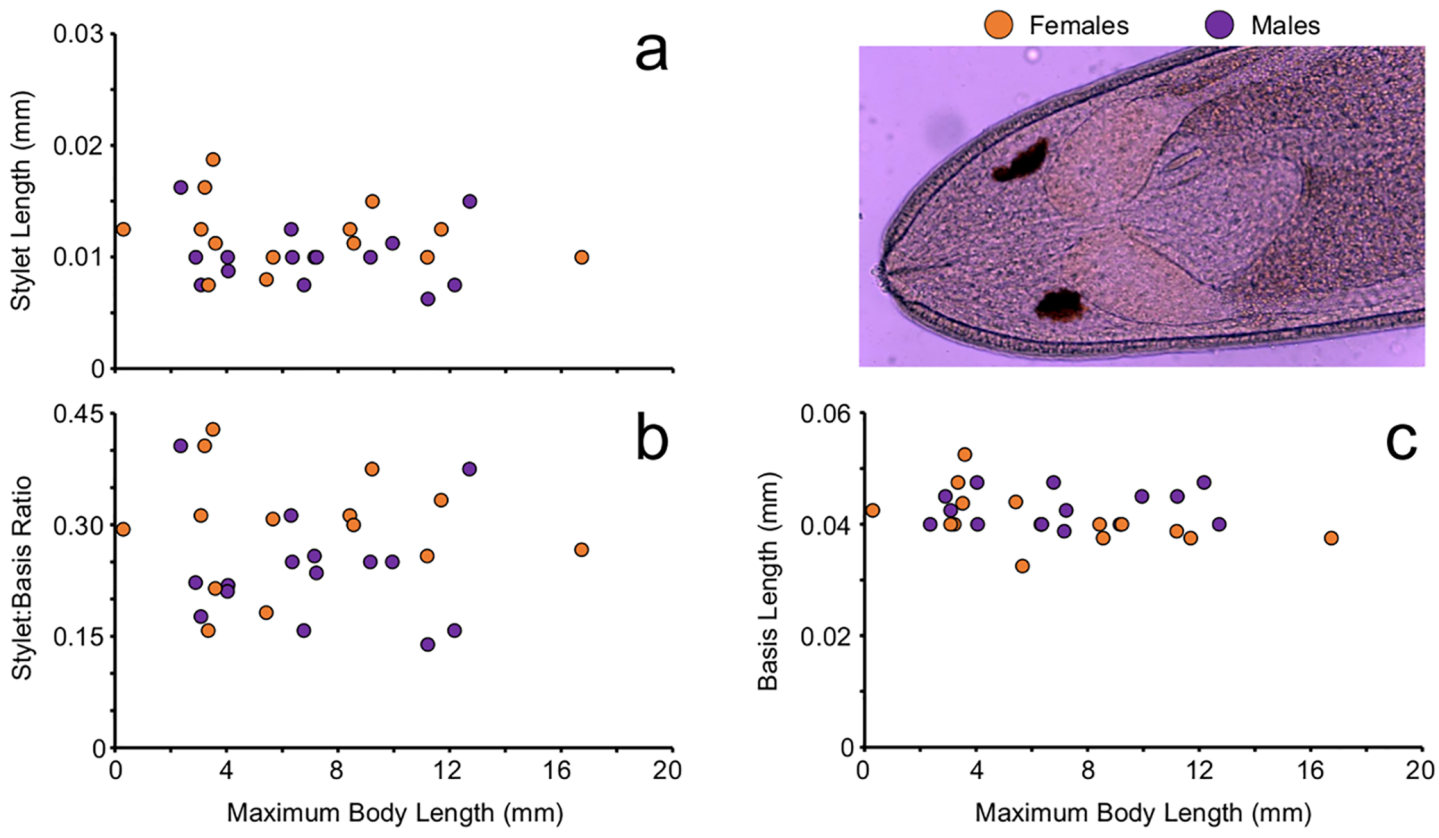
Relationship between stylet characteristics and maximum body length [MBL] for male and female worms. (a) relationship between MBL and stylet length [SL] for both sexes. (b) relationship between MBL and the stylet:basis ratio [SBR] for both sexes. (c) relationship between MBL and basis length [BL] for both sexes. In all instances there was no impact of sex on these relationships.

We explored the relationship between stylet characteristics (stylet length, basis length, and stylet:basis ratio) and maximum body length for male and female *C*. *conanobrieni* and found no significant correlation between the variables in all instances [stylet length (b = -0.00008, R = 0.0102, t = -9.289, df = 1,14, P = 0.5157; b = -0.0001, R = 0.0412, t = -16.947, df = 1,13, P = 0.6096, for males and females respectively), basis length (b = 0.0001, R = 0.0161, t = -32.1232, df = 1,14, P = 0.7253; b = -0.0005, R = 0.2340, t = -39.347, df = 1,13, P = 0.1768, for males and females respectively), and stylet:basis ratio (b = -0.00022, R = 0.0101, t = -7.912, df = 1,14, P = 0.5232; b = -0.0002, R = 0.0001, t = -14.795, df = 1,13, P = 0.9359, for males and females respectively)] (Fig 6). Overall, the size of the stylet remained the same irrespective of worm body size.

Furthermore, when looking to see if sexual dimorphism played a role in the size of these stylet characters, we found no evidence of such. An ANCOVA looking at the relationship between sex, body length, and stylet length showed there was no effect of sex (F = 2.4368, df = 1, 28, P = 0.1311) or body length (F = 0.711, df = 1, 28, P = 0.4071) and the interaction term was not significant (F = 0.1085, df = 1, 28, P = 0.7446). This indicates that neither body size nor sex has an impact on the length of the stylet. An ANCOVA looking at the relationship between worm body size, sex, and basis length showed there was no effect of sex (F = 1.8877, df = 1, 28, P = 0.1817) or of body size (F = 0.3416, df = 1, 28, P = 0.5642) and the interaction term was not significant (F = 1.1812, df = 1, 28, P = 0.2875). The ANCOVA looking at the interaction between sex, body size, and stylet:basis ratio showed that there was no effect of sex (F = 3.1379, df = 1, 28, P = 0.0887), there was no effect of body size (F = 0.2949, df = 1, 28, P = 0.5919), and that the interaction term was not significant (F = 0.3873, df = 1, 28, P = 0.5393). The above means, that regardless of body size or sex, worms exhibit the same stylet:basis relationship.

### Phylogenetic analysis

**Fig 7:**
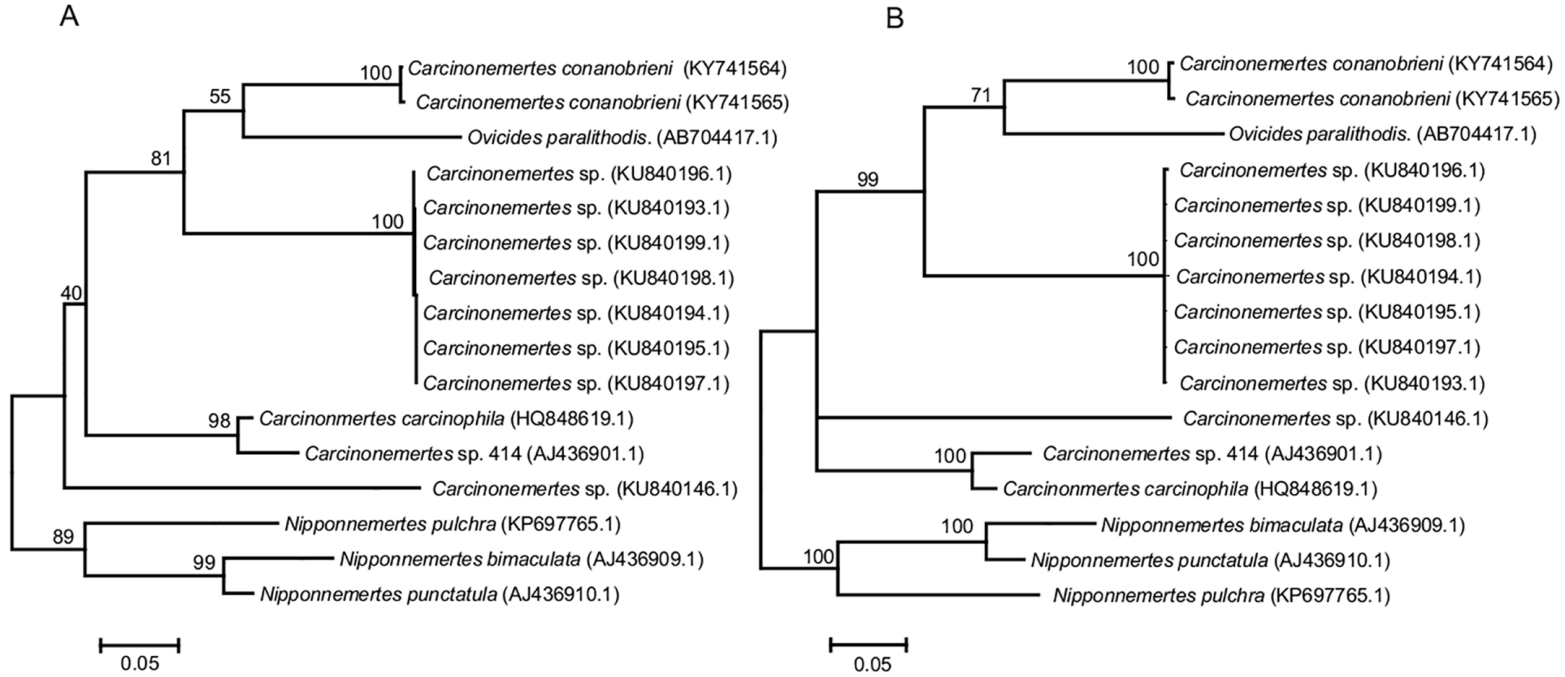
Maximum likelihood and Bayesian inference phylogenetic trees. A maximum likelihood tree (a) and a Bayesian inference tree (b) both depict the phylogenetic relationship between our species (*C*. *conanobrieni*) and all *Carcinonemertes* species where COI sequences were available. Outgroup species used include *Ovicides* sp., *Nipponnemertes punctatus*, *Nipponnemertes bimactulata*, and *Nipponnemertes pulchra*. Both trees show clear separation between our species and all other species used in the anaylses. Accession numbers for GenBank are listed in parenthesis next to the species names.

Both maximum likelihood and Bayesian inference analyses clustered our two samples of *C*. *conanobrieni* together (100 and 1.0 bootstrap and support values from ML and BI analyses, respectively) and separated them from all other available COI sequences for other *Carcinonemertes* spp, *Ovicides* sp, and the selected outgroups. This indicates that *C*. *conanobrieni* is in fact a genetically distinct entity from all other species for which there are COI sequences available (Fig 7). The genetic distance (p-value) between the two *Carcinonemertes conanobrieni* specimens was only 0.003 while the distance between *Carcinonemertes conanobrieni* specimens and representatives from other species in the phylogenetic analysis was much greater, ranging from 0.038 to 0.158.

### Behavior

Mature specimens were found either free-roaming or ensheathed within the egg mass of host lobsters (Fig 1). Immature specimens were found either free-roaming or encapsulated next to a single lobster embryo (Fig 4). When removed from the egg mass and placed in a Petri dish filled with seawater, worms would either attach themselves to the glass interior or swim along the top of the water. Worms could produce copious amounts of mucus while in the Petri dish and were sometimes found grouped together on floating ‘sheets’ of mucus at the water’s surface. Specimens could move relatively quickly around the edges of the petri dish, though when mechanically disturbed, they could be slow to respond. The most common response to mechanical disturbance was to move the body either forward or backwards to evade the forcep’s tip. Sometimes, a specimen would wrap itself around the forceps tip and adhere to it with mucus. When placed into the MgCl_2_ solution worms would quickly coil into a spiral and produce enough mucus to coat the entire body (this layer had to be gently stripped away with forceps prior to measurements being taken). The worm specimens were fragile and great care had to be taken not to tear them when moving or adjusting them with forceps.

### Ecology

This worm is symbiotic with the Caribbean spiny lobster, *P*. *argus*, and may even be considered an obligatory parasite or micropredator since all life stages were observed within the brood masses of its hosts, and because worms have been shown to diminish reproductive output in infected lobsters [32] (Figs 1 and 2). Mature female worms lay mucus encased eggs throughout the lobster broods; these egg cases have a smooth surface and can be either spherical in shape or long strings entwined through the lobster’s setae (Fig 2). These worms also produce a mucus sheath that covers the body of the worm and is wound throughout the lobster’s setae (Fig 1). This sheath is usually the same length of the worm or slightly longer, it is also decorated across its surface with protruding ‘hooks.’

### Host and parasite distribution

*Carcinonemertes conanobrieni* were found on gravid *P*. *argus* females from all sites in the Florida Keys that the lobsters were sampled. Worms were almost exclusively found within the brood masses of their hosts, and observed only once on the abdomen of a female that had hatched her embryos between the time of collection and parasite examination. Though the presence of the worm was exclusive to the abdominal brooding space and its content, other body parts of lobsters of different sex and ontogenetic stages cannot be ruled out as potential microhabitats also capable of harboring worms at this time.

## Taxonomic remarks

The species described above aligns with the diagnosis of both Carcinonemertidae [27, 43] and *Carcinonemertes* [27, 44, 45] as being gonochoric with the absence of accessory stylets and pouches. In the following, we discuss the differences between *C*. *conanobrieni* and all previously described species within the genus *Carcinonemertes*. *Carcinonemertes conanobrieni* exhibits distinct differences from *Carcinonemertes* species that may be considered sympatric (Table 2), *Carcinonemertes* species that have been found to infect other lobster species (Table 3), as well as all other described *Carcinonemertes* species (S1 Table).

**Table 2:** Comparison of morphological and ecological traits of *Carcinonemertes conanobrieni* sp. nov. to sympatric species (*Carcinonemertes carcinophila carcinophila*, *Carcinonemertes carcinophila immunta*, and *Carcinonemertes pinnotheridophila*).

Character		*C*. *conanobrieni*		*C*. *c*. *carcinophila*		*C*. *c*. *imminuta*		*C*. *pinnotheridophila*	
		Male	Female	Male	Female	Male	Female	Male	Female
Worm Body Color		Translucent White to Cream	Translucent White to Pale Orange	Yellowish-Orange, Pale Reddish, Rose Pink, Brick Red		Whitish	Reddish	Off-White or Tan	Orange-Red
Body Length		2.35–12.71 mm	0.296–16.73 mm	6.0–70.0 mm		8.68 mm (average)	16.55 mm (average)	2.3 mm (max)	8.4 mm (max)
Body Width		0.157–0.331 mm	0.246–3.02 mm	----		0.214 mm (average)	0.22 mm (average)	----	
Infestation Site		Egg Mass		Gill lamelle, Egg Mass		Gill lamelle, Egg Mass		Branchial Chamber, Egg Mass	
Ocelli Characters	Number	2		2		4 / 2		No Ocelli	
	Color	Bright Orange to Red		Black		Yellowish-Brown, Brown, Black			
	Shape	Irregular (Cup or Elliptical)		Elliptical		Irregular			
Distance from Eyes to Head		0.106–0.229 mm	0.083–0.211 mm	----		----	0.135 mm	----	
Distance between Eyes		0.043–0.111 mm	0.054–0.143 mm	----		----	0.200 mm	----	
Stylet Length		0.006–0.016 mm	0.008–0.019 mm	0.006–0.012 mm		0.006–0.0095 mm		0.0066 mm	0.008 mm
Basis Length		0.039–0.048 mm	0.033–0.053 mm	0.020–0.030 mm		0.019–0.023 mm		0.0181 mm	0.016 mm
Stylet:Basis Ratio		0.139–0.407	0.158–0.429	0.316–0.400		0.0461		0.365	0.5
Mucus Sheath		Yes (ornamented)		Yes (ornamented)		Yes		Yes	
Egg Sheath Shape		----	Long Strands or Spherical Cases	----	Long Strands	----	Long Strands	----	Spherical Cases

**Table 3:** Comparison of morphological and ecological traits of *Carcinonemertes conanobrieni* sp. nov. to *Carcinonemertes* species that have been found on other species of spiny lobster (*Carcinonemertes wickhami* and *Carcinonemertes australiensis*).

Character		*C*. *conanobrieni*		*C*. *wickhami*		*C*. *australiensis*	
		Male	Female	Male	Female	Male	Female
Worm Color		Translucent White to Cream	Translucent White to Pale Orange	Pinkish-White	Orange	Translucent White	
Body Length		2.35–12.71 mm	0.296–16.73 mm	5–18 mm	10–30 mm	7 mm	
Body Width		0.157–0.331 mm	0.246–3.02 mm	0.400 mm		1 mm	
Infestation Site		Egg Mass		Egg Mass		Egg Mass	
Ocelli Characters	Number	2		2		2	
	Color	Bright Orange to Red		Black		Black	
	Shape	Irregular (Cup or Elliptical)		Cup		----	
Distance from Eyes to Head		0.106–0.229 mm	0.083–0.211 mm	0.163 mm	0.145 mm	----	
Distance between Eyes		0.043–0.111 mm	0.054–0.143 mm	0.162 mm	0.257 mm	----	
Stylet Length		0.006–0.016 mm	0.008–0.019 mm	0.019–0.200 mm		0.015–0.018 mm	
Basis Length		0.039–0.048 mm	0.033–0.053 mm	0.036–0.042 mm		0.040 mm	
Stylet:Basis Ratio		0.139–0.407	0.158–0.429	0.476–0.528		0.375–0.45	
Mucus Sheath		Yes (ornamented)		Yes (ornamented)		No	
Egg Sheath Shape		----	Long Strands or Spherical Cases	----	Long Strands	----	Not Reported

*Carcinonemertes carcinophila carcinophila*, a sympatric species, differs from *C*. *conanobrieni* in terms of maximum body length, ocelli characteristics, mucus sheath ornamentation, shape of egg cases, host specificity, and infestation site (Table 2). *Carcinonemertes c*. *carcinophila* has a reported maximum body length of 70 mm [44] while the maximum for *C*. *conanobrieni* is 16.73 mm. The two ocelli of *C*.*c*. *carcinophila* are described as being elliptical in shape and black in color [27], while those of *C*. *conanobrieni* vary both in shape (irregular, circular, elliptical) and color (bright orange to rusty red). *Carcinonemertes carcinophila carcinophila* produces mucus sheaths that display lapilli cells across the sheath [45]. In contrast, *C*. *conanobrieni* has hooks protruding along the sheath. Eggs laid by *C*.*c*. *carcinophila* are distributed throughout the brood mass of host crabs in long strings [28]. *Carcinonemertes conanobrieni* instead lays eggs in the brood mass of the host both in long strings and in nearly perfectly spherical sacs. Furthermore, *C*.*c*. *carcinophila* does not appear to be host specific, with it having been found infecting at least 28 different crustacean hosts [27, 28]. *Carcinonemertes carcinophila carcinophila* also infects both male and female crab hosts and may be found within the gill chambers or on the brood masses of female crabs [46]. Thus far, *C*. *conanobrieni* has only been found on gravid (or recently gravid) female *P*. *argus*, and only within the brooding space, although additional studies are needed to confirm these preliminary observations.

A second sympatric species, *Carcinonemertes carcinophila imminuta*, differs from *C*. *conanobrieni* in terms of body length, body width, ocelli characteristics, mucus sheath ornamentation, number of ovaries, shape of egg cases, host specificity, and infestation site (Table 2). The maximum reported body length for *C*. *c*. *immunuta* is 35 mm for females and 16 mm for males [27] while the maximum body length of *C*. *conanobrieni* is 16.73 mm for females and 12.71 for males. Maximum body width of *C*.*c*. *imminuta* females is 0.22 mm and males is 0.214 mm [27]; *C*. *conanobrieni* has a maximum body width of 3.02 mm for females and 0.331 mm for males. Furthermore, while adult *C*.*c*. *imminuta* have 2 irregular shaped eyes colored with yellowish-brown, brown, or black and larvae have 4 irregular shaped eyes of the same color [27], *C*. *conanobrieni* has two irregular shaped eyes both as a larva and as an adult. *Carcinonemertes carcinophila imminuta* and *C*. *conanobrieni* both produce ornamented mucus sheaths, though *C*.*c*. *imminuta* displays lapilli cells [45] while *C*. *conanobrieni* has hooks arranged along the sheath. The largest measured female *C*.*c*. *imminuta* has a reported 370 ovaries [27] while *C*. *conanobrieni* averages 87.4 ± 43.6 ovaries. Eggs laid by *C*.*c*. *imminuta* are positioned throughout the brood mass of host crabs in long strings. *Carcinonemertes conanobrieni* also lays eggs in the brood mass of the host in long strings, but also in nearly perfect spherical sacs. *Carcinonemertes carcinophila imminuta* does not exhibit host specificity and has been found on multiple crustacean hosts [27] and is reported on both male and females, as well as on immature and mature crabs. As stated above *C*. *conanobrieni* so far has only been found on gravid or recently gravid female *P*. *argus*.

*Carcinonemertes pinnotheridophila*, a third sympatric species, differs from *C*. *conanobrieni* in terms of body length, ocelli characteristics, mucus sheath, basis length, stylet:basis ratio, and in host of choice (Table 2). *C*. *pinnotheridophila* has a smaller maximum body size reported at 8.4 mm for females and 2.3 mm for males [47], while *C*. *conanobrieni* has a maximum body size of 16.73 mm in females and 12.71 mm in males. *Carcinonemertes pinnotheridophila* lack ocelli at any life stage, while *C*. *conanobrieni* have two ocelli throughout their lives. Furthermore, while *C*. *pinnotheridophila* does secrete a mucus sheath, this sheath is not ornamented, *C*. *conanobrieni* secretes a mucus sheath ornamented with hooks. The length of the basis is 0.016 mm for female *C*. *pinnotheridophila* and 0.0181 mm for males, which is considerably smaller than what we have found for *C*. *conanobrieni* with a mean basis length of 0.041 **±** 0.005 mm for females and 0.043 **±** 0.003 for male worms. The stylet:basis ratio of *C*. *pinnotheridophila* was 0.5 for females and 0.365 for males, while in *C*. *conanobrieni*, the ratio was 0.296 **±** 0.078 mm for females and 0.241 **±** 0.087 for males, which is smaller. While both *C*. *pinnotheridophila* and *C*. *conanobrieni* are seemingly host-specific, they differ in their chosen hosts. *Carcinonemertes pinnotheridophila* are found in the egg masses of brooding *Pinnixa chaetopterana* as well as in the branchial chamber of non-brooding females. Though *C*. *pinnotheridophila* is only reported to infect *Pinnixia chaetopterana*, it shares characteristics that are similar to the undescribed *Carcinonemertes* spp. that infect *Zaops ostreum* (in North Carolina) and *Austinixa gorei* (in Florida) [47]. *Carcinonemertes conanobrieni* has thus far only been found in association with brooding female *P*. *argus*.

The newly described species also exhibits characteristics that distinguish it from the two other species of *Carcinonemertes* that have been found to infect lobsters (Table 3). One such species, *Carcinonemertes wickhami*, differs from *C*. *conanobrieni* in terms of body size and sexual dimorphism, ocelli characteristics, distance between ocelli, distance from ocelli to the tip of the head, and mucus sheath production (Table 3). *Carcinonemertes wickhami* displays noticeable sexual dimorphism with females having a range of body lengths from 10–30 mm while males range from 5–18 mm [23]. On the other hand, *C*. *conanobrieni* displays little sexual dimorphism with females exhibiting a range in size from 0.292–16.73 mm and males from 2.35–12.71 mm. *C*. *wickhami* have two eyes that are black in color and cup shaped [23] while those of *C*. *conanobrieni* vary both in shape (irregular, circular, elliptical) and color (bright orange to rusty red). Female *C*. *wickhami* have eyes that are 0.257 mm apart and 0.145 mm to the tip of the head, males have eyes that are 0.162 mm apart and 0.163 mm from the tip of the head. The mean distance between the eyes for *C*. *conanobrieni* is 0.087 ± 0.025 mm for females and 0.077 ± 0.022 mm for males which is more narrow than *C*. *wickhami*; the distance from the eyes to the tip of the head was 0.166 ± 0.041 mm for females and 0.175 ± 0.031 mm for males which is larger than for *C*. *wickhami*. C*arcinonemertes wickhami* also produces lapilli-covered mucus sheaths which differ from the hook-ornamented sheaths of *C*. *conanobrieni*. As we have reported for *C*. *conanobrieni*, *C*. *wickhami* worms may also be found on the egg-bearing pleopods of female lobsters and additionally at the base of the uropods; with no reports from male lobsters and juveniles lobsters [16, 23].

*Carcinonemertes australiensis* infects the spiny lobster *Panulirus cygnus*, and differs from *C*. *conanobrieni* in both body width and mucus sheath production (Table 3). The reported body dimensions of a single *C*. *australiensis* individual are 7 mm long and 1 mm wide [24]. *Carcinonemertes conanobrieni* displays a mean width of 0.540 ± 0.647 mm in females and 0.235 ± 0.0420 mm in males, both of which are smaller than what is reported for *C*. *australiensis*. In contrast to *C*. *conanobrieni*, there is no report of a mucus sheath being produced by *C*. *australiensis*. In agreement with other lobster-infecting species, *C*. *australiensis* has been reported to inhabit only the egg masses of brooding females [16, 24].

S1 Table shows the differences between *C*. *conanobrieni* and all remaining described species of *Carcinonemertes*. With a mean body length and range of 7.03 ± 3.41 mm (2.35–12.71 mm) for male worms and a mean body length and range of 6.12 ± 4.32 mm (0.292–16.73 mm) for female worms, *C*. *conanobrieni* is considerably smaller than *Carcinonemertes mitsurii* (max 100 mm [males] and max 300 mm [females]) [27]. *Carcinonemertes conanobrieni* is reported as being larger than *Carcinonemertes divae* (2.6 ± 0.2 mm (males) and 2.6 ± 0.1 mm (females)) [45], *Carcinonemertes caissarum* (2.0 ± 0.3 mm (males) and 5.5 ± 1.0 mm (females)) [45], *Carcinonemertes regicides* (1.6 mm (males) and 2.1 mm (females)) [23, 29], *Carcinonemertes kurisi* (1.8 ± 0.1 mm (males) and 4.5 ± 0.3 mm (females)) [29], and *Carcinonemertes tasmanica* (1.9 ± 0.7 mm (males) and 5.6 ± 1.3 mm (females)) [29]. Furthermore, *C*. *conanobrieni* does not exhibit the relatively high sexual size dimorphism (males << females) that is reported for *C*. *caissarum*, *C*. *kurisi*, *C*. *tasmanica*, and *C*. *mitsukurii* [27, 29, 45].

*Carcinonemertes conanobrieni* varies in body color from a translucent white to a pale orange, this aligns with many species within the genus *Carcinonemertes*, but does differ from the body colors of *C*. *caissarum*, where males have a red spot on the posterior end, *C*. *errans* (pink to reddish orange) [30, 48], *C*. *regicides* (pink, red-orange, and dull orange) [43], *C*. *epialti* (bright orange to reddish-yellow) [27, 45], *C*. *kurisi* females (dark orange to red-pink) [29], and *C*. *tasmanica* (red) [29].

With a stylet basis mean length and range of 0.043 ± 0.003 mm (0.039–0.048 mm) for male *C*. *conanobrieni* and 0.041 ± 0.005 mm (0.033–0.0053 mm) for female *C*. *conanobrieni*, our species has a basis that is longer than almost every other species. The only exception to this is *C*. *regicides* which has a stylet basis length of 0.0405 mm [27, 43].

The mucus sheath with decorative hooks that is produced by *C*. *conanobrieni* is very different from the sheaths that are reported for other species of *Carcinonemertes*. *Carcinonemertes regicides* forms a mucus sheath that is not decorated and breaks very easily when manipulated [43], *C*. *kurisi* and *C*. *tasmanica* both produce distinctive corkscrew shaped sheaths [29], and *C*. *sebastianensis*, *C*. *caissarum*, and *C*. *diavae* produce sheaths covered in lapilli [45].

Finally, *C*. *conanobrieni* differs from all species mentioned in S1 Table in that they have been found infecting the Caribbean spiny lobster, *P*. *argus*. Thus far, *C*. *conanobrieni* is the only species of *Carcinonemertes* reported to infect *P*. *argus*. The species of *Carcinonemertes* found in S1 Table have all been found in crab hosts, and host specificity differs in these species. *C*. *errans* has been shown to be extremely host-specific, while *C*. *mitsukurii* [27], C. *divae* [45], *C*. *caissarum* [45], *C*. *sebastianensis* [45], *C*. *kurisi* [45, 29], and *C*. *tasmanica* [29] have all been reported on a single host. *C*. *epialti* shows host preference, but is not specific to a single crab species.

As previously stated, the considerable amount of morphological homogeneity in the genus *Carcinonemertes* has in the past made species identification both difficult and at times unclear [24, 27, 45]. This difficulty arises from small size of the worms, the similarities in ecology and morphology driven by a parasitic lifestyle, and the ambiguity that comes from distinguishing closely linked morphological structures [45]. In addition, the use of these ambiguous structures for initial identification can make future identification more difficult. We feel that some structures that have been used in the past for species differentiation are not well suited for the task. For instance, using the shape of the posterior and anterior ends of the worms (as in [45]) may lead to confusion in some cases. We found that the shape of the ends of *C*. *conanobrieni* showed some variation as a result of how relaxed the specimen was, if the worm was fully extended or not, and the amount of water present under the cover-slip. The ocelli, a character often used in species descriptions [24, 27, 48] is another example of a character with too much ambiguity. We found that there was variation in the color of the eyes of *C*. *conanobrieni*, more light led to eyes that were bright orange in color, and less light to a rusty-red. This difference in eye coloration of many of *Carcinonemertes* species can most likely be attributed to different light intensities. This should be taken into consideration when using eye color as a diagnostic character. Furthermore, the shape of the eyes varied with the orientation of the worm and with whether or not a cover-slip was present. However, the presence or absence of ocelli and the number of ocelli are both clearly quantitative measures that should continue to be used.

Based on our observations and what has been to this point reported, there are a number of morphological and ecological characteristics that can offer clear distinctions between species. Some of these include the external mucus sheath, stylet/basis characteristics, body size, number of ovaries, and host specificity. Because of the stark differences that exist for the mucus sheath, whether a sheath is produced or not, the presence or absence of lapilli cells, the presence of decorative hooks, and shape, it can be reliable means of distinguishing one species from another. Interestingly, we found that the sizes of the stylet and its basis did not change with worm body size for *C*. *conanobrieni*; if this is true for other species of *Carcinonemertes* then the stylet and basis can be very reliable for species differentiation. Since their sizes are not impacted by growth after sexual maturity, any significant differences that exist can clearly define a species. Body size (there is a wide reported range of sizes) and sexual size dimorphism (whether or not it occurs) can also be useful tools in separating species. Furthermore, we agree with Santos et al. [45] in that adding more measurements of practical morphological characters (i.e. number of ovaries, distance from ovaries/testes to the head, distance between the ocelli) when describing species within this group of worms will help to improve both quality description as well as the understanding of the observed extant diversity. By increasing the number of externally visible morphological characters that are measured the description and differentiation of species should become much more attainable, allowing researchers to tackle the current abundance of undescribed nemertean worms [49]. Furthermore, the addition of genetic characters will be exceedingly helpful in future studies looking to resolve the group’s phylogeny (see, 49).

## Supporting information

S1 Table Comparison of morphological and ecological traits of *Carcinonemertes conanobrieni* sp. nov. to *Carcinonemertes* species that are considered non-sympatric and are found on non-lobster hosts. Morphological measurements from *Carcinonemertes mitsukurii*, *Carcinonemertes divae*, *Carcinonemertes caissarum*, *Carcinonemertes sebastianensis*, *Carcinonemertes coei*, *Carcinonemertes errans*, *Carcinonemertes regicides*, *Carcinonemertes humesi*, *Carcinonemertes epialti*, *Carcinonemertes kurisi*, and *Carcinonemertes tasmanica* taken from the literature for a comparative table.
(DOCX)

S2 Table Raw morphological measurements for *Carcinonemertes conanobrieni*, sp. nov. (XLSX)

S1 Text COI sequences of two *Carcinonemertes conanobrieni* sp. nov. specimens used for phylogenetic analysis. (TXT)
