# Supplementary material for: A new species of Carcinonemertes, Carcinonemertes conanobrieni sp. nov. (Nemertea: Carcinonemertidae), an egg predator of the Caribbean spiny lobster, Panulirus argus
Source: PLoS One. 2017 May 5;12(5):e0177021. doi: 10.1371/journal.pone.0177021 (PMC5419566; doi:10.1371/journal.pone.0177021)
Supplement: S1 Table — Morphological measurements from Carcinonemertes mitsukurii, Carcinonemertes divae, Carcinonemertes caissarum, Carcinonemertes sebastianensis, Carcinonemertes coei, Carcinonemertes errans, Carcinonemertes regicides, Carcinonemertes humesi, Carcinonemertes epialti, Carcinonemertes kurisi, and Carcinonemertes tasmanica taken from the literature for a comparative table. (DOCX) [file pone.0177021.s001.docx]

Supplementary Table 1: Comparison of morphological and ecological traits of *Carcinonemertes conanobrieni* sp. nov. to *Carcinonemertes* species that are considered non-sympatric and are found on non-lobster hosts: *Carcinonemertes mitsukurii*, *Carcinonemertes divae*, *Carcinonemertes caissarum*, *Carcinonemertes sebastianensis*, *Carcinonemertes coei*, *Carcinonemertes errans*, *Carcinonemertes regicides*, *Carcinonemertes humesi*, *Carcinonemertes epiliti*, *Carcinonemertes kurisi*, *Carcinonemertes tasmanica*.

| Character | | ***C. conanobrieni*** | | ***C. mitsukurii*** | | ***C. divae*** | | ***C. caissarum*** | | ***C. sebastianensis*** | | ***C. coei*** | | | ***C. errans*** | | | ***C. regicides*** | | | ***C. humesi*** | | | | ***C. epilti*** | | ***C. kurisi*** | | ***C. tasmanica*** | |
| --- | --- | --- | --- | --- | --- | --- | --- | --- | --- | --- | --- | --- | --- | --- | --- | --- | --- | --- | --- | --- | --- | --- | --- | --- | --- | --- | --- | --- | --- | --- |
|  |  | Male | Female | Male | Female | Male | Female | Male | Female | Male | Female | Male | Female | | Male | Female | Male | | Female | Male | | Female | | Male | | Female | Male | Female | Male | Female |
| Worm Body Color | | Translucent White to Cream | Translucent White to Pale Orange | Light Brown, Yellow, Milky White | | Cream | Translucent White, Orange | Cream w/ Red Spot at Posterior End | Translucent White to Cream | Translucent White | | Yellowish-White | | | Pink, Reddish, Orange, White Spots | | Pink to Dull Orange | | Red-Orange, Pink, Dull Orange | Translucent, Off-White | | | | Bright Orange, Reddish, Yellow | | | Whitish-Pink | Dark Orange, Reddish-Pink | Red | |
| Body Length | | 2.35-12.71 mm | 0.296-16.73 mm | 100 mm | 300 mm | 2.6 +/- 0.2 mm | 2.6 +/- 0.1 mm | 2.0 +/- 0.3 mm | 5.5 +/- 1 mm | 6.2 +/- 1.4 mm | 7.0 +/- 4.0 mm | 6 mm | | | 4.0-6.0 mm | | 1.6 mm | | 2.1 mm | ---- | | | | ---- | | | 1.8 +/- 0.1 mm | 4.5 +/- 0.3 mm | 1.9 +/- 0.7 mm | 5.6 +/- 1.3 mm |
| Body Width | | 0.157-0.331 mm | 0.246-3.02 mm | 0.5 cm | | 0.316 +/- 0.032 mm | 0.332 +/- 0.017 | 0.400 +/- 0.025 mm | 0.282 +/- 0.020 mm | 0.362 +/- 0.028 mm | 0.405 +/- 0.062 mm | 0.5 mm | | | ---- | | ---- | | 0.236 mm | ---- | | | | ---- | | | ---- | | ---- | |
| Infestation Site | | Egg Mass | | Egg Mass, Gills | | Egg Mass, Abdomen, Arthrodial Membranes, Pereopods | | Egg Mass, Abdomen, Gonopores, Pleopods (Arthrodial Membranes and Setae) | | Egg Mass, Abdomen, Setae, Pleopods | | Egg Mass | | | Egg Mass | | Gills, Abdomen, Axillae, Egg Mass | | | Gills, Abdomen, Egg Mass | | | | Gills, Abdomen, Egg Mass | | | Egg Mass | | Setae of Pleopods, Abdomen | |
| Ocelli Characters | Number | 2 | | 2 | | 2 | | 2 | | 2 | | 2 to 4 | | | 2 | | 2 | | | 2 | | | | 2 | | | 2 | | 2 | |
|  | Color | Bright Orange to Red | | Black | | Black | | Black | | Brown | | Dark brown, Black | | | Black, Brown | | ---- | | | Reddish-Brown | | | | Black, Brown | | | Brown | | Black, Brown | |
|  | Shape | Irregular (Cup or Elliptical) | | Spots | | Irregular | | Round, Cup-Like | | Circular, Elliptical | | Irregular | | | Irregular | | Cup-Like | | | Irregular | | | | Irregular | | | Irregular | | Irregular | |
| Distance from Eyes to Head | | 0.106-0.229 mm | 0.083-0.211 mm | ---- | 0.200 mm | 0.106 +/- 0.010 mm | 0.143 +/- 0.0095 mm | 0.106 +/- 0.007 mm | 0.110 +/- 0.018 mm | 0.112 +/- 0.011 mm | 0.110 +/- 0.018 mm | ---- | | | ---- | | ---- | | 0.131 mm | ---- | | | | 0.136-0.200 mm | | | 0.075 mm | | ---- | |
| Distance between Eyes | | 0.043-0.111 mm | 0.054-0.143 mm | ---- | 0.135 mm | 0.096 +/- 0.003 mm | 0.103 +/- 0.004 mm | 0.107 +/- 0.009 mm | 0.112 +/- 0.011 mm | 0.110 +/- 0.005 mm | 0.131 +/- 0.014 mm | ---- | | | ---- | | ---- | | 0.149 mm | ---- | | | ---- | | | | 0.122 mm | | ---- | |
| Stylet Length | | 0.006-0.016 mm | 0.008-0.019 mm | 0.008 mm | | 0.010 +/- 0 mm | 0.0010 +/- 0.001 mm | 0.008 +/- 0.001 mm | 0.008 +/- 0.001 mm | 0.011 +/- 0 mm | 0.009 +/- 0.001 mm | 0.0087 mm | | | 0.011 +/- 0.002 mm | | ---- | | 0.0172 mm | 0.007-0.008 mm | | | | 0.021-0.023 mm | | | 0.005 mm | | 0.0067 mm | |
| Basis Length | | 0.039-0.048 mm | 0.033-0.053 mm | 0.027 mm | | 0.028 +/- 0.001 mm | 0.025 +/- 0.001 mm | 0.021 +/- 0.001 mm | 0.022 +/- 0.001 mm | 0.025 +/- 0.001 mm | 0.022 +/- 0.001 mm | 0.0227 mm | | | 0.0351 +/- 0.003 mm | | ---- | | 0.0405 mm | 0.030-0.032 mm | | | | 0.010-0.015 mm | | | 0.020 mm | | 0.0133 mm | |
| Stylet:Basis Ratio | | 0.139 -0.407 | 0.158-0.429 | 0.296 | | 0.371 | 0.387 | 0.372 | 0.0378 | 0.431 | 0.416 | 0.382 | | | 0.314 | | ---- | | | 219-167 | | | | 0.333-0.500 | | | ---- | | 50 : 40.8 | |
| Mucus Sheath | | Yes (ornamented) | | Yes | | Yes (Lapilli Cells) | | Yes (Lapilli Cells) | | Yes (Lapilli Cells) | | No | | | No | | Yes (Fragile, Not Decorated) | | | ---- | | | | ---- | | | Yes (Distinctive Corkscrew) | | Yes (Distinctive Corkscrew) | |
| Egg Sheath Shape | | ---- | Long Strands or Ovular Cases | ---- | Tubes | ---- | Long Strands | ---- | Long Strands | ---- | Long Strands | ---- | | Cylindrical Strands | ---- | Cylindrical Strands | ---- | | Long Strands | ---- | | | | ---- | | | ---- | Cylindrical Strands | ---- | |
